# Collagen-Derived Di-Peptide, Prolylhydroxyproline (Pro-Hyp): A New Low Molecular Weight Growth-Initiating Factor for Specific Fibroblasts Associated With Wound Healing

**DOI:** 10.3389/fcell.2020.548975

**Published:** 2020-11-27

**Authors:** Kenji Sato, Tomoko T. Asai, Shiro Jimi

**Affiliations:** ^1^Division of Applied Biosciences, Graduate School of Agriculture, Kyoto University, Kyoto, Japan; ^2^Department of Food Science and Nutrition, Faculty of Human Life and Environment, Nara Women's University, Nara, Japan; ^3^Central Laboratory for Pathology and Morphology, Department of Pathology, Faculty of Medicine, Fukuoka University, Fukuoka, Japan

**Keywords:** collagen, fibroblast, Pro-Hyp, collagen peptide, wound healing, p75NTR, mesenchymal stem cells

## Abstract

Many cells and soluble factors are involved in the wound healing process, which can be divided into inflammatory, proliferative, and remodeling phases. Fibroblasts play a crucial role in wound healing, especially during the proliferative phase, and show heterogeneity depending on lineage, tissue distribution, and extent of differentiation. Fibroblasts from tissue stem cells rather than from healthy tissues infiltrate wounds and proliferate. Some fibroblasts in the wound healing site express the mesenchymal stem cell marker, p75NTR. In the cell culture system, fibroblasts attached to collagen fibrils stop growing, even in the presence of protein growth factors, thus mimicking the quiescent nature of fibroblasts in healthy tissues. Fibroblasts in wound healing sites proliferate and are surrounded by collagen fibrils. These facts indicate presence of new growth-initiating factor for fibroblasts attached to collagen fibrils at the wound healing site, where the collagen-derived peptide, prolyl-hydroxyproline (Pro-Hyp), is generated. Pro-Hyp triggers the growth of p75NTR-positive fibroblasts cultured on collagen gel but not p75NTR-negative fibroblasts. Thus, Pro-Hyp is a low molecular weight growth-initiating factor for specific fibroblasts that is involved in the wound healing process. Pro-Hyp is also supplied to tissues by oral administration of gelatin or collagen hydrolysate. Thus, supplementation of gelatin or collagen hydrolysate has therapeutic potential for chronic wounds. Animal studies and human clinical trials have demonstrated that the ingestion of gelatin or collagen hydrolysate enhances the healing of pressure ulcers in animals and humans and improves delayed wound healing in diabetic animals. Therefore, the low molecular weight fibroblast growth-initiating factor, Pro-Hyp, plays a significant role in wound healing and has therapeutic potential for chronic wounds.

## Introduction

Cutaneous wound healing is a complex and highly regulated process with many cells and soluble factors working together during this process (Han and Ceilley, 2017; Stunova and Vistejnova, 2018; Cañedo-Dorantes and Cañedo-Ayala, 2019). The cutaneous wound healing process is generally divided into three overlapped phases: inflammatory, proliferative, and remodeling or maturation phases. The inflammatory phase starts shortly after hemostasis and is triggered by the activation of adhered platelets. Chemokines and cytokines, which are released from the activated platelets and damaged tissue, recruit neutrophils and monocytes to the wound sites (Golebiewska and Poole, 2015). Neutrophils, which appear in the wound 4 h after injury and decrease during the subsequent weeks, eliminate pathogens by phagocytosis and the release of bactericidal reactive oxygen species and peptides (Brinkmann et al., 2004). The infiltration of monocytes starts the second day and the monocytes are changed to macrophages. Macrophages increase and reach maximum levels during the proliferative process. Macrophages scavenge tissue debris and the remaining neutrophils by phagocytosis and secrete protein growth factors and cytokines that promote proliferation and migration of the cells involved in wound healing (Wynn and Barron, 2010; Ploeger et al., 2013). Circulating lymphocytes migrate to the wound healing sites early after injury and remain up to the last phase. Approximately 3 days after the initial injury, the proliferative phase starts. This phase is responsible for the lesion closure. Fibroblasts infiltrate to the wound sites and proliferate and produce extracellular compounds such as collagen, which form the basis of granulation tissue. The granulation tissue provides the basis for re-epithelization and some fibroblasts differentiate into myofibroblasts, initiating wound contraction (Tomasek et al., 2002). To supply nutrients to the granulation tissue, the induction of angiogenesis occurs (Greaves et al., 2013). After ~2–3 weeks, the proliferative phase is followed by the remodeling or maturation phase to achieve tensile strength through contraction, reorganization, degradation, and resynthesis of the extracellular matrix (Xue and Jackson, 2015). The cells involved in the previous phases are removed by apoptosis. Thus, the granulation tissue is gradually remodeled and changed to scar tissue consisting of less cellular and vascular components and rich in collagen fibers, resulting in an ~70–80% tensile strength of the uninjured skin (Stunova and Vistejnova, 2018). Aberrant wound healing may result in a chronic wound or non-healing wound, which is a burden to the patient, caregiver, and medical system. Aberrant wound healing is often occurs under diabetic and malnutrition conditions (Han and Ceilley, 2017).

In wound healing process, fibroblasts play a significant role especially during the proliferative phase. Fibroblasts migrate, proliferate, and produce extracellular matrix compounds in response to protein growth factors, such as epidermal growth factor, insulin, interleukin-1β, tumor necrosis factor-α, transforming growth factor-β1, platelet-derived growth factor, and fibroblast growth factors (Ejiri et al., 2015; Cañedo-Dorantes and Cañedo-Ayala, 2019). In cell culture systems, fibroblasts proliferate on plastic substrate in the presence of fetal bovine serum (FBS), which is rich in protein growth factors and fibronectin. The removal of low molecular weight compounds from FBS does not affect the growth of fibroblasts on the plastic substrate (Asai et al., 2020a). Fibroblasts have to attach to the substrates for survival. In tissues, fibroblasts attach to collagen fibrils directly using collagen receptors such as α1β1 and α2b1 integrins (Zeltz and Gullberg, 2016) and indirectly via fibronectin using α5β1 integrin (Wu et al., 1993). In cell culture systems using non-coated culture plates, fibroblasts are attached to the plate surface via fibronectin, which is present in FBS and promote cell proliferation (Hayman and Ruoslahti, 1979). However, some researchers have used collagen gel-coated plates for the cultivation of fibroblasts (Yoshisato et al., 1985; Nishiyama et al., 1989; Kono et al., 1990; Shigemura et al., 2009; Asai et al., 2020a,b). The collagen solution becomes to be polymerized at neutral pH after incubation at 37°C. During this process, collagen molecules assemble into collagen fibrils (Yoshisato et al., 1985). Fibroblasts that are attached to the collagen fibrils get in a quiescent state even in the presence of FBS. The denatured collagen also forms gel but does not assemble into the fibrils, and cannot inhibit the growth of fibroblasts. A higher concentration of collagen gels induce a robust inhibitory effect on fibroblast proliferation (Yoshisato et al., 1985; Kono et al., 1990). Therefore, the inhibitory effect of collagen gel on fibroblast growth cannot be solely attributed to softness of collagen gels compared to plastic plate, while it is known that extracellular mechanical stress affect the growth and differentiation of cells, including fibroblasts (Jansen et al., 2017). Thus, fibroblasts growth is controlled not only by protein growth factors but also by interaction with collagen fibrils. Growth regulatory mechanisms of fibroblast settled on collagen fibrils are thus vital for understanding the *in vivo* wound healing process. Nevertheless, this topic has received limited attention many years up to now.

Normal fibroblasts present in a quiescent state all over the connective tissues in the body under physiological condition. The suppression of proliferation of fibroblast cultured on collagen gel could therefore mimic the quiescent characteristics of fibroblasts in healthy tissue (Yoshisato et al., 1985; Kono et al., 1990). However, fibroblasts in collagen matrixes start to proliferate after wounding, in which, additional factor(s) are required for initiating fibroblast proliferation. However, rational information is still limited for the fibroblasts under such context. Our group have previously addressed; after total skin excision in mice, collagen di-peptide, prolyl-hydroxyproline (Pro-Hyp), which is generated in the granulation tissue after wounding (Jimi et al., 2017) and Pro-Hyp can act as a trigger for growth initiation in the fibroblasts cultured on collagen gel (Asai et al., 2020a,b; Shigemura et al., 2009).

The present study does not aim to review cutaneous wound healing processes comprehensively. Instead, the present mini review is thus focusing especially on the function of a new low molecular weight growth-initiating factor, Pro-Hyp, in wound healing process and discuss its therapeutic potential for chronic wounds in the end.

## Generation of Collagen-Derived Di-Peptides In Tissues

Collagen is a major extracellular matrix protein and has a triple helical domain and small globular domains. Collagen forms a molecular family consisting of different gene products. Collagen molecular species are referred to as “Type” with Roman numerals (Van der Rest and Garrone, 1991). Types I, II, III, V, and XI collagens form collagen fibrils and are referred to as fibril-forming collagens. Other collagen types are classified as non-fibrillar collagen, which play essential biological functions; however, their contents are far lower than that of the fibrillar collagens (Van der Rest and Garrone, 1991). Type I collagen is the primary collagen in the skin, bone, and tendons. Type III and V collagens co-exist with Type I collagen. Type II collagen is the primary collagen in cartilage with minor Type XI collagen. Collagen molecules consist of three subunits referred to as the α chain. Each α chain is designated by Roman numerals (Type) and Arabic numerals (subunit number). The major collagen molecules in the skin, Types I and III collagens, are designated as [α1(I)]_2_α2(I) and [α1(III)]_3_, respectively.

Collagen consists of post-translationally modified amino acids, hydroxyproline (Hyp), and hydroxylysine (Hyl), which play significant roles in stabilizing the triple helix structure and the inter- and intra-molecular cross-links of collagen molecules, respectively (Rappu et al., 2019). Prolyl residues especially in the Y position of Gly-X-Y motif in collagen are frequently changed to a hydroxyprolyl residue by prolyl hydroxylase. Each collagen subunit has ~80–100 Hyp residues depending on the collagen type and animal species. The Gly-Pro-Pro motif is most abundant in Gly-X-Pro- motifs (43, 23, and 37 repeats) in the predicted sequences of human mature α1(I), α2(I), and α1(III) chains, respectively (ExPASy, Entry numbers P02452, P08123, P02461). Thus, the Gly-Pro-Hyp motif is present most abundantly in major collagens in the skin.

The triple helix structure of collagen resists most proteinases, except for collagenases such as matrix metalloproteinases (MMPs)-1 and−8 (Jabłońska-Trypuć et al., 2016). Collagenase fragments with a triple helix structure can collapse to a denatured form with a globular structure at body temperature. This denatured form of collagen fragments can be degraded by other endoproteinases and exopeptidases into amino acids and oligopeptides (Sunada and Nagai, 1983).

Pro-Hyp increases in the blood of patients who have bone metastasis cancer (Mazzi et al., 1996; Inoue et al., 2001). The Pro-Hyp motif is most abundant in major collagens, and Pro-Hyp resists human plasma peptidases (Iwai et al., 2005). Therefore, Pro-Hyp is liberally released by the degradation of endogenous collagen presented in tissues.

**Figure 1:**
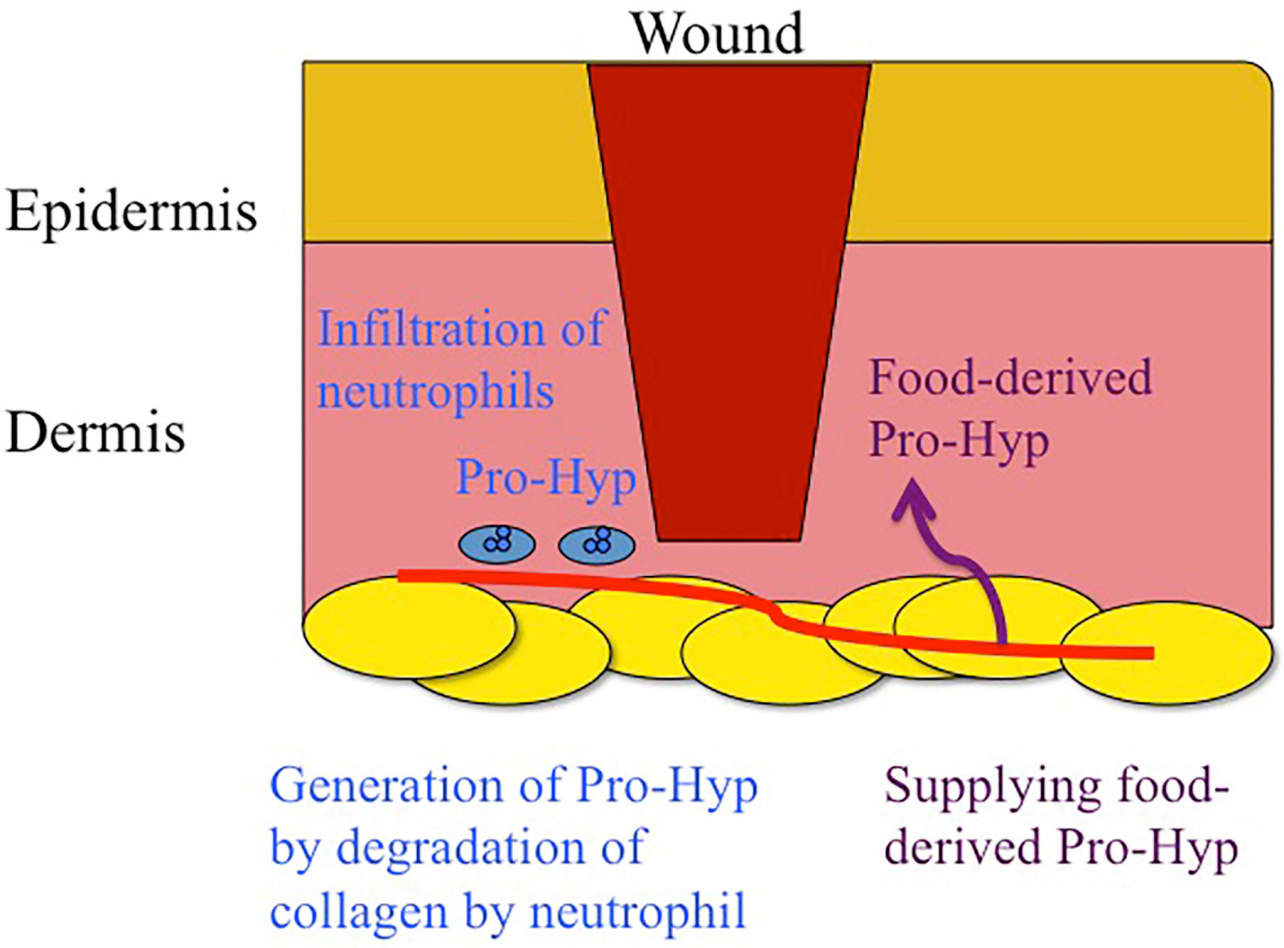
Schematic drawing of generation of Pro-Hyp by degradation of endogenous collagen by neutrophils and supplementation of food derived Pro-Hyp around wound.

Pro-Hyp was also found to be generated in the granulation tissue at wound healing site of mouse (C57BL/6) skin after excision and did not increase in the skin of the healthy tissues of the same animal (Jimi et al., 2017). The infiltration of NIMP-R14- and MMP-8-positive neutrophils into the granulation tissue was also observed. The generation of Pro-Hyp in the granulation tissue of the skin of the db/db mouse, which has a mutation in the gene encoding the leptin receptor and is susceptibile to obesity, insulin resistance, and Type 2 diabetes, was significantly lower than that in the wild-type mouse (C57BL/6) during all periods of wound healing (day 1–8) (Jimi et al., 2017). Decreased infiltration of NIMP-R14- and MMP-8-positive neutrophils into the granulation tissue and lower levels of blood granulocyte colony-stimulating factor (G-CSF) were also observed in db/db mice. The topical application of recombinant human G-CSF onto the wound of the db/db mice increased the number of infiltrated neutrophils and Pro-Hyp in the granulation tissue (Jimi et al., 2017). Therefore, neutrophils play a significant role in the generation of Pro-Hyp in granulation tissue by the excretion of collagenase (MMP-8) as illustrated in Figure 1.

Pro-Hyp, Leu-Hyp, and Gly-Pro-Hyp were significantly increased in the mouse ear with dermatitis, whereas these peptides did not increase in other ear without dermatitis in the same animal (Kusubata et al., 2015; Sato et al., 2019). Among these, Pro-Hyp was the most abundant; however, isotope-labeled Pro-Hyp was specifically cleaved in the ear with dermatitis. Thus, very rapid synthesis and degradation of Pro-Hyp occurs in tissues under inflammation.

Taken together, these data suggest that collagen-derived tri- and di-peptides are generated by the degradation of major collagen in tissues under inflammation where extracellular matrix remodeling occurs. Pro-Hyp is a major compound owing to its high peptidase resistance and the abundance of Pro-Hyp motifs in the major collagen types I and III in the skin.

## Food-Derived Collagen Peptides In Body

Collagen in food material is denatured by heat treatment and converted to gelatin. Gelatin is soluble in hot water; therefore, hot water can extract gelatin from animal, bird, and fish skin as well as their bones. Gelatin is easily degraded by food-grade proteases, which produce peptides with molecular weights of 1,000–5,000 Da (Iwai et al., 2005; Osawa et al., 2018). This gelatin hydrolysate is referred to as collagen hydrolysate or collagen peptide, which is used as a food ingredient.

At least 12 collagen-derived peptides, e.g., Pro-Hyp, Hyp-Gly, Pro-Gly, Glu-Hyp, Ala-Hyp, Ile-Hyp, Leu-Hyp, Phe-Hyp, Ser-Hyp-Gly, Ala-Hyp-Gly, Gly-Pro-Hyp, and Pro-Hyp-Gly, have been identified in human blood after the ingestion of collagen hydrolysate and meat rich in collagen (Iwai et al., 2005; Ohara et al., 2007; Ichikawa et al., 2010; Shigemura et al., 2011, 2018; Asai et al., 2019). Hydroxyproline containing peptides have been shown to significantly increase after the ingestion of collagen hydrolysate in a dose-dependent manner (Shigemura et al., 2014). In some cases, it can reach ~100 μM or more (Iwai et al., 2005; Ohara et al., 2007), which is much higher than the previously reported values for food-derived peptides in human blood (Matsui et al., 2002; Foltz et al., 2007). In all cases, Pro-Hyp was most abundant in human blood plasma, accounting for ~50% of the total collagen peptides in the human blood. Thus, the ingestion of collagen hydrolysate can supply collagen peptides, which have the same structure as endogenous collagen peptides in wound healing sites and inflammatory tissues (Figure 1).

Approximately 24 h after the ingestion of collagen peptides, the plasma Pro-Hyp level returned to its initial level, with some of the Pro-Hyp excreted in the urine (Taga et al., 2019). Animal study using [^14^C] Pro-Hyp demonstrated that radioactivity was observed in rat skin fibroblasts, femur chondrocytes, and synovial cells 30 min after the ingestion of [^14^C] Pro-Hyp (Kawaguchi et al., 2012). Thin layer chromatography analysis revealed that [^14^C] Pro-Hyp in cartilage was metabolized to other modified peptides and amino acids; however, it was not identical to proline. Some of the orally administered Pro-Hyp was incorporated into these cells and further metabolized; however, the metabolites formed were not identified.

## Pro-Hyp–Low Molecular Weight Fibroblast Growth-Initiating Factor

In general, collagen-coated plates are utilized for acquiring stabilized growth and differentiation in cultured cells. Fibroblasts attached to collagen fibrils-assembled collagen gel, however, stop their growth even in the presence of FBS (Yoshisato et al., 1985; Kono et al., 1990; Shigemura et al., 2009; Asai et al., 2020a,b). However, contradict results have been reported (Nishiyama et al., 1989; Asai et al., 2020a). To clarify the contradiction, Asai et al. (2020a) demonstrated that in fibroblasts cultured on collagen gels, the cells could proliferate in the medium containing an FBS, although, when low molecular weight compounds <6,000 Da were eliminated from the FBS, fibroblast growth could cease on collagen gel, however, the fractionalized FBS could not stop cellular proliferation on a plastic substrate. The medium was supplemented with conventional low molecular weight nutrients such as amino acids, glucose, vitamins, and minerals. Thus, the compound(s) responsible for the switch of proliferation on/off on collagen gel culture are present in the low molecular weight fraction in FBS, while growth factors and attachment factors including fibronectin still remained in the fractionated FBS. Using the fractionated FBS, the effect of Pro-Hyp, an endogenous and food-derived collagen di-peptide, was examined on the growth of fibroblasts cultured on collagen gel. Consequently, Pro-Hyp (200 μM) stimulated proliferation of fibroblasts cultured on the collagen gel (Asai et al., 2020a,b). This small peptide is, therefore, a low molecular weight fibroblast growth-initiating factor (LMW-FGIF). Pro-Hyp was also contained in relatively high levels (up to approximately 45 μM) in commercially available FBS, but it depended upon suppliers, brands, and lots (Asai et al., 2020a). The presence of Pro-Hyp in FBS at different levels has been a stumbling block for the detection and identification of LMW-FGIF using the cell culture system. There is the possibility that other collagen-derived peptides could be classified as LMW-FGIF, although their contents might be lower than those of Pro-Hyp. The authors recommend that the low molecular weight compounds should be removed from commercially available FBS or use of serum-free and defined media when seeking other LMW-FGIF when using a cell culture system.

## Heterogeneity of Skin Fibroblasts In Association With Wound Healing

**Figure 2:**
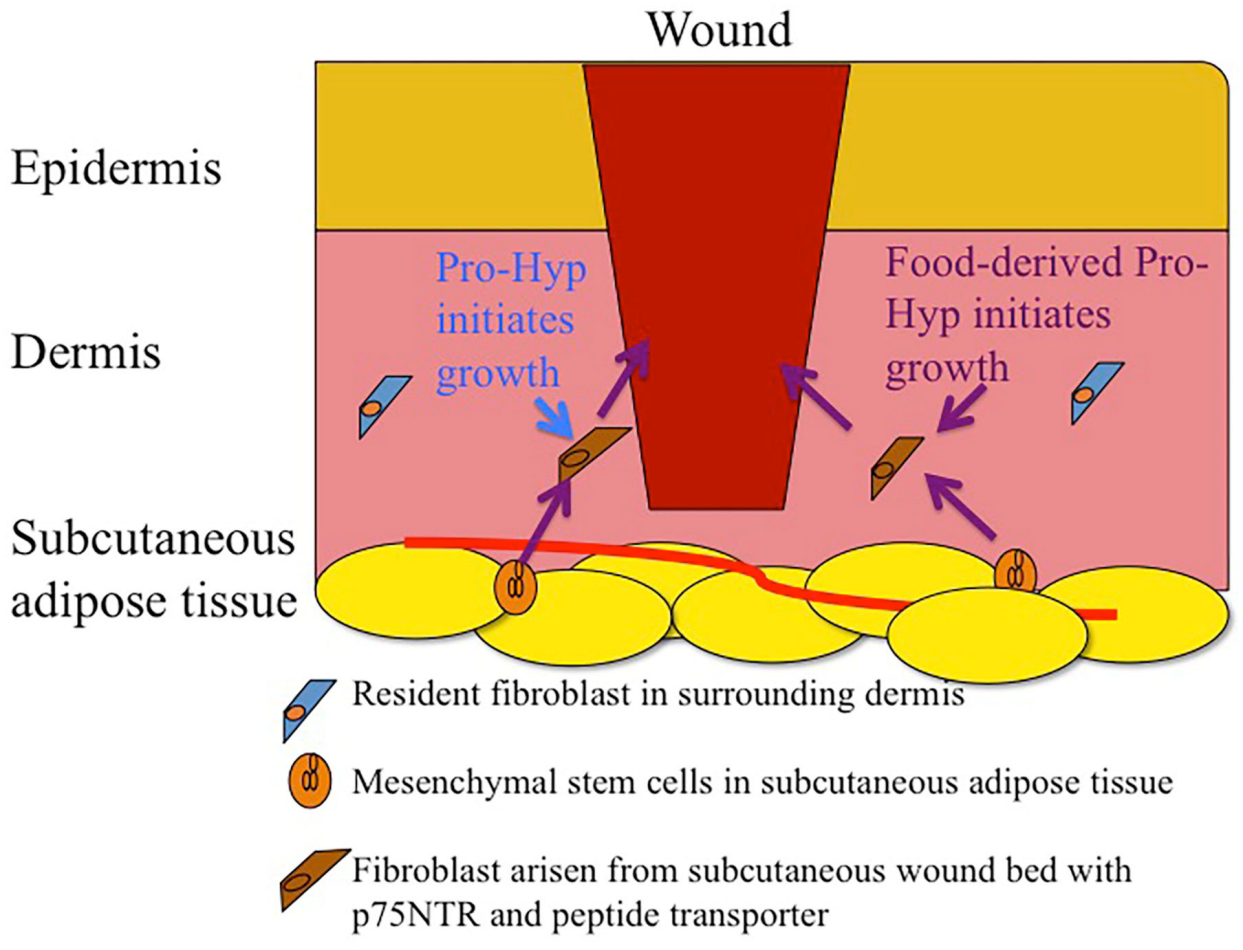
Schematic drawing of infiltration of fibroblasts arisen from subcutaneous wound bed mesenchymal stem cells rather than fibroblasts in healthy dermis. Endogenous and food-derived Pro-Hyp initiates growth of the fibroblasts arisen from subcutaneous wound bed.

Fibroblasts are an ill-defined cell groups. Many cell surface markers for fibroblasts have been proposed. These markers are expressed temporally on fibroblasts depending on the ontogeny process and their distribution in tissues (Driskell et al., 2013). Some markers, such as platelet-derived growth factor receptor and some cytoskeletons, are present in all fibroblasts, while these markers are also present in non-fibroblast cells. Thus, there is no universal cell surface marker that is specific for fibroblasts. Adult skin fibroblasts show heterogeneity and plasticity (des Jardins-Park et al., 2018; Stunova and Vistejnova, 2018; Cañedo-Dorantes and Cañedo-Ayala, 2019). Dermal fibroblasts have been shown to arise from at least two different lineages: the upper dermal and lower lineage. In the adult skin, mesenchymal stem cells are located in hair follicles (Ge et al., 2016) and subcutaneous adipose tissue (Yamamoto et al., 2007). Fibroblasts, which migrate to the wound site and form granulation tissue during the proliferative phase, have been shown to arise from the subcutaneous wound bed (lower lineage) rather than from the surrounding healthy dermis (Rossio-Pasquier et al., 1999; Geer et al., 2004; Driskell et al., 2013). Thus, fibroblasts differentiated from mesenchymal stem cells rather than resident fibroblasts in the healthy dermis play a significant role in the proliferative phase of the wound healing process as illustrated in Figure 2. The International Society for Cellular Therapy (2006) defined mesenchymal stem cells as being able to adhere to plastic plates, differentiate into osteoblasts, adipocytes, or other cell types; and express CD73, CD90, and CD 105 (Dominici et al., 2006). However, fibroblasts in some cell lines from different tissues also express these marker proteins (Denu et al., 2016). Thus, low-affinity nerve growth factor receptor (p75NTR or CD271) has been used as mesenchymal stem cell markers in bone marrow, adipose tissue, and the dermis (Tomellini et al., 2014; Álvarez-Viejo et al., 2015; Pincelli, 2017). p75NTR was initially discovered in neuronal cells (Fabricant et al., 1977). p75NTR-positive cells are present around the bulge region of hair follicles of healthy mouse, which co-express Sox-2, nestin, and S100β, all makers for nerve-terminal-associated neural crest precursor cells (Johnston et al., 2013). Some of these cells have been suggested to be associated with axons that are sprouted into the regenerating dermis after skin injury (Johnston et al., 2013). However, p75NTR is also expressed in various non-neuronal cell types, such as fibroblasts and macrophages, under inflammatory conditions (Trim et al., 2000; Nakamura et al., 2006; Palazzo et al., 2012; Meeker and Williams, 2014). These studies have shown that p75NTR-positive cells play an important role in wound healing and accumulate in the granulation tissue, whereas only a small number of p75NTR-positive cells are observed in healthy skin (Iwata et al., 2013).

**Figure 3:**
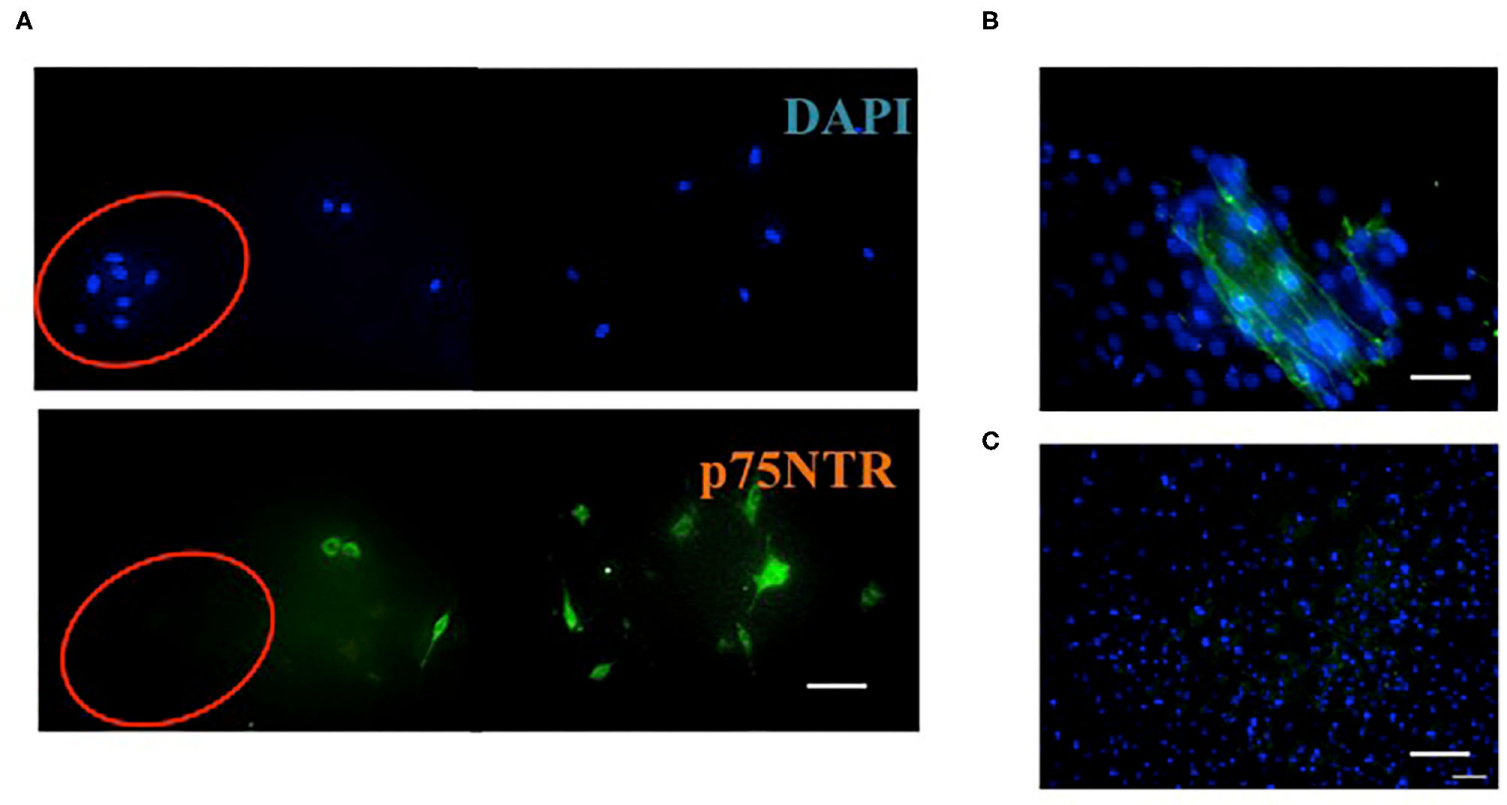
Immunocytochemistry of cells on the outside of mouse skin after incubation for 1 day **(A)**, 2 weeks **(B)**, and 4 weeks **(C)**. A: Cells were stained with DAPI (blue) or antibodies against p75NTR (green). Cells in the red circle are keratinocytes; the rest are fibroblasts. **(B,C)** Fibroblasts were stained with DAPI (blue) and antibodies against p75NTR (green). Scale bar = 50 μm. This figure was adapted with permission (Asai et al., 2020b).

Huang et al. (2013) reported that fibroblast-like cells, which were obtained from trypsinized human skin, did not express p75NTR after 4**–**5 days of cultivation in DMEM containing 10% FBS. In contrast, as shown in Figure 3, fibroblasts, which migrated from the piece of skin in the same medium, expressed p75NTR and p75NTR-positive fibroblasts declined after few day cultivation (Asai et al., 2020b). Thus, fibroblasts differentiated from mesenchymal stem cells temporally expressed p75NTR but rapidly lost p75NTR after growth.

**Figure 4:**
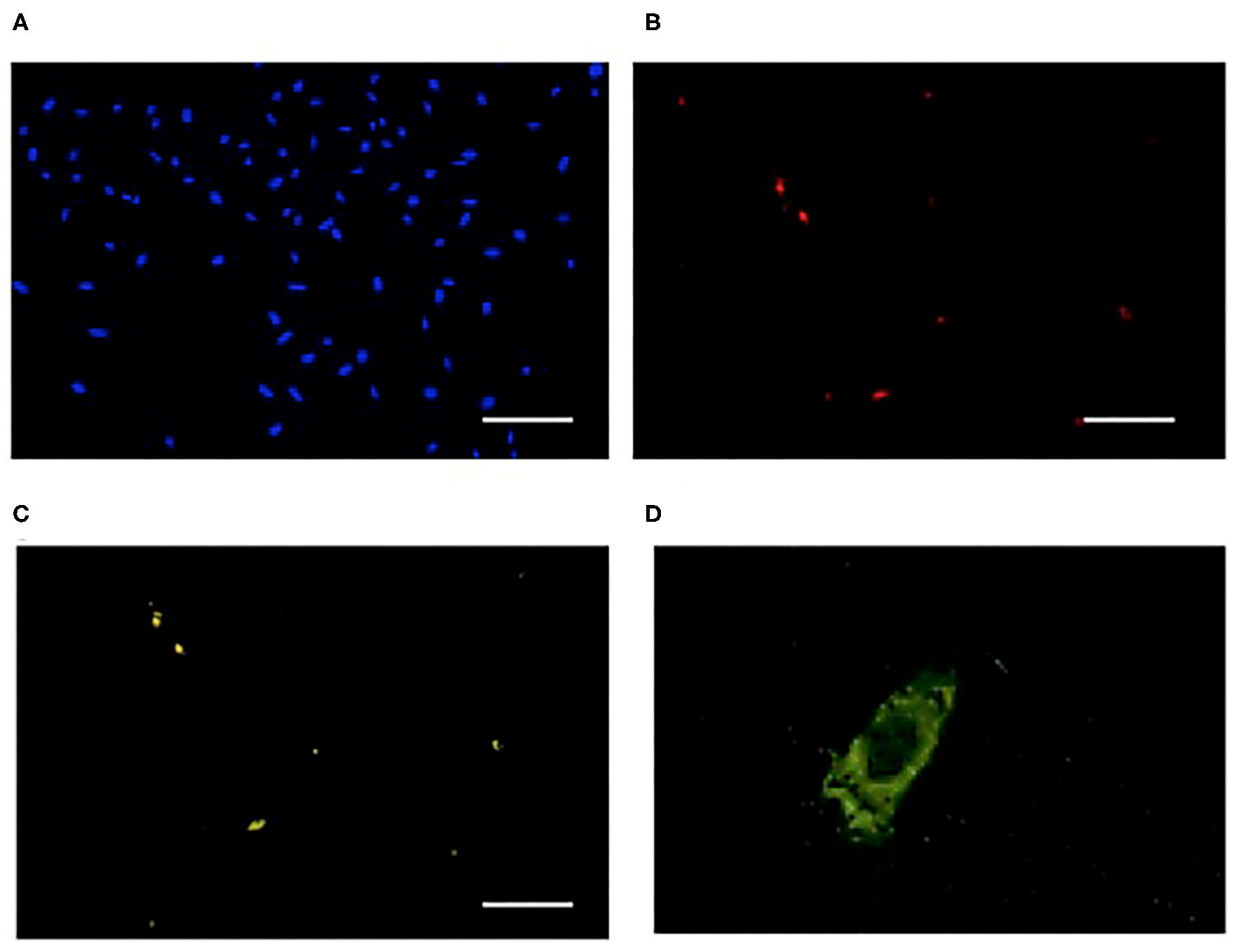
Incorporation of FITC-labeled Pro-Hyp into fibroblasts. FITC-labeled Pro-Hyp was specifically incorporated into 75NTR-positive fibroblasts. Fibroblasts outside of the skin after 4 days of incubation were used. Cells were stained with DAPI in **(A)**, antibody against p75NTR (red) in **(B)**, fluorescence of FITC-labeled Pro-Hyp (green) in **(C)**. Scale bar = 50 μm. High magnification picture of fibroblast incorporating FITC-labeled Pro-Hyp in **(D)**. This figure was adapted with permission (Asai et al., 2020b).

Pro-Hyp, LMW-FGIF, proliferates p75NTR-positive fibroblasts cultured on the collagen gel but not p75NTR-negative fibroblasts (Asai et al., 2020b). Liquid chromatography-tandem mass spectrometry analysis revealed that Pro-Hyp was incorporated into mouse skin fibroblasts; however, the incorporation was suppressed after prolonged cultivation (Asai et al., 2020b). The incorporation of Pro-Hyp into p75NTR-positive and -negative fibroblasts was examined by using fluorescein isothiocyanate (FITC)-labeled Pro-Hyp because FITC-labeled and non-labeled di-peptides pass through the same peptide transporter 1 (PepT1) (Abe et al., 1999). The fluorescence of the FITC-labeled Pro-Hyp was only observed in the p75NTR-positive fibroblasts (Figures 4A–C) and the fluorescence was weak on the nuclei (Figure 4D). Thus, the FITC-labeled Pro-Hyp was incorporated into the cytosol rather than being bound on the cell surface (Asai et al., 2020b). p75NTR-positive fibroblasts specifically incorporate Pro-Hyp, which might trigger the proliferation of fibroblasts cultured on collagen gel. Even the primary cultured fibroblasts, however, changed their nature within a few days, which makes it difficult to elucidate the response of fibroblasts to LMW-FGIF and the interaction between extracellular matrixes using a cell culture system. The expression of p75NTR and the ability to incorporate di-peptides are useful biomarkers for distinguishing fibroblast subpopulations in cell culture systems.

## Influence of The Supplementation of Collagen Peptides on Wound Healing

Diabetic patients often suffer from chronic wounds, the mechanism of which is not fully understood (Han and Ceilley, 2017). The generation of Pro-Hyp in the granulation tissue of a db/db mouse, a diabetic model animal, was smaller than that in a normal mouse (Jimi et al., 2017). Pro-Hyp is crucial for the proliferation of p75NTR-positive fibroblasts cultured on collagen gel. Some animal studies have also demonstrated that the oral administration of fish gelatin and collagen hydrolysate improved the delayed wound healing in diabetic rats and mice, respectively (Zhang et al., 2011; Xiong et al., 2020). Therefore, supplementation of collagen hydrolysate or gelatin has therapeutic potential for chronic wounds in diabetic patients. To the best of our knowledge, human clinical trials have not been undertaken to prove the therapeutic effect of collagen hydrolysate on chronic wounds in diabetic patients.

Pressure ulcers are another chronic wound with several animal studies suggesting that supplementation with collagen hydrolysate improves the healing of pressure ulcers (Nakao et al., 2013). Human clinical trials using placebo controls have demonstrated that supplementation with collagen hydrolysate enhances healing of pressure ulcers (Lee et al., 2006; Sugihara et al., 2015, 2018; Yamanaka et al., 2017). The Japanese Society of Pressure Ulcers Guideline Revision Committee (2016) has cited collagen hydrolysate in their therapeutic guidelines. In these studies, Pro-Hyp, which is derived from orally administered collagen hydrolysate, was considered to play a crucial role in enhancing the healing of pressure ulcers. Sugihara et al. (2018) demonstrated that low molecular weight collagen hydrolysate (average molecular weight: 1,200 Da) that was rich in Pro-Hyp exerted better therapeutic effects on patients with pressure ulcers than the conventional collagen hydrolysate (5,000 Da). The ingestion of collagen hydrolysate with low molecular weight has been shown to increase blood collagen peptide levels compared to that with high molecular weight (Ichikawa et al., 2009). However, different blood collagen peptide levels were observed after ingestion of the same dose of collagen hydrolysates with similar average molecular weights made from different sources (Ohara et al., 2007). Therefore, the therapeutic effect of collagen hydrolysate depends on its source and preparation method, and formulated collagen hydrolysate, which can provide reproducible Pro-Hyp levels in the blood after ingestion, is required for wound healing therapy.

Food-derived Pro-Hyp circulates in the blood system and is potentially delivered to all tissues. However, an *in vitro* study indicated that p75NTR-negative fibroblasts in healthy tissue did not respond to Pro-Hyp (Asai et al., 2020b). No adverse effects due to abnormal proliferation of fibroblasts have been observed after long-term administration of collagen hydrolysate in healthy volunteers (Shigemura et al., 2018) and pressure ulcer patients (Sugihara et al., 2015, 2018; Yamanaka et al., 2017). Thus, supplementation with collagen hydrolysate does not generate severe adverse effects on non-wound tissue.

An animal study suggested that the supplementation of collagen hydrolysate has the potential to promote healing of wounds after cesarean section (Wang et al., 2015). Yasueda et al. (2016) reported that the supplementation of collagen hydrolysate reduced hospital stays after colon cancer surgery. These preliminary reports suggest that supplementation with collagen hydrolysate might enhance the healing of surgical wounds. However, the effects of food-derived Pro-Hyp on the growth of cancer cells and cancer-associated fibroblasts have not been examined. In addition, there is limited data on the effect of the ingestion of collagen hydrolysate on carcinogenesis in animal models. The oral administration of a collagen fraction to hamsters with chemo-induced pancreatic duct carcinoma (0.4% in the diet) for 50 days did not show exacerbation (Kitahashi et al., 2006). The effects of Pro-Hyp on carcinogenesis in other animal models should be examined before it is used to promote surgery recovery in cancer patients.

## Conclusion

Human clinical trials and animal studies have demonstrated that the ingestion of gelatin and collagen hydrolysate enhances wound healing, especially diabetes-induced chronic wounds and pressure ulcers. After the ingestion of collagen hydrolysate, Pro-Hyp increases in human blood. Pro-Hyp is also generated by the degradation of endogenous collagen in wound healing sites as summarized in Figure 1. Endogenous and food-derived Pro-Hyp can enhance wound healing by stimulating the growth of p75NTR-positive fibroblasts in the wound healing site without adverse effects on healthy tissue because it does not significantly affect quiescent p75NTR-negative fibroblasts in healthy tissue (Figure 2). The small collagen peptide, Pro-Hyp, is a low molecular weight growth-initiating factor for fibroblasts and plays a crucial role in wound healing by initiating the proliferation of fibroblasts with mesenchymal stem cell marker, p75NTR and peptide transporter(s).

## Author Contributions

KS: conceptualization and writing—original draft. SJ: supervising, reviewing, and editing. TA: reviewing and editing. All authors contributed to the article and approved the submitted version.

## Conflict of Interest

The authors declare that the research was conducted in the absence of any commercial or financial relationships that could be construed as a potential conflict of interest.
